# Comparison of the effects of dexmedetomidine and propofol on hypothermia in patients under spinal anesthesia: a prospective, randomized, and controlled trial

**DOI:** 10.7150/ijms.72754

**Published:** 2022-05-16

**Authors:** Minhye Chang, Sung-Ae Cho, Seok-Jin Lee, Tae-Yun Sung, Choon-Kyu Cho, Young Seok Jee

**Affiliations:** Department of Anesthesiology and Pain medicine, Konyang University Hospital, Konyang University College of Medicine, Daejeon, Korea

**Keywords:** Hypothermia, Propofol, Dexmedetomidine, Anesthesia, spinal, Sedatives, Temperature

## Abstract

**Background:** Redistribution hypothermia caused by vasodilation during anesthesia is the primary cause of perioperative hypothermia. Propofol exerts a dose-dependent vasodilatory effect, whereas dexmedetomidine induces peripheral vasoconstriction at high plasma concentrations. This study compared the effects of dexmedetomidine and propofol on core temperature in patients undergoing surgery under spinal anesthesia.

**Methods:** This prospective study included 40 patients (aged 19-70 years) with American Society of Anesthesiologists Physical Status class I-III who underwent elective orthopedic lower-limb surgery under spinal anesthesia. Patients were randomly allocated to a dexmedetomidine or propofol group (*n* = 20 per group). After induction of spinal anesthesia, patients received dexmedetomidine (loading dose: 1 μg/kg over 10 min; maintenance dose: 0.2-0.7 μg/kg/h) or propofol (loading dose: 75 μg/kg over 10 min; maintenance dose: 12.5-75 μg/kg/min). The doses of sedatives were titrated to maintain moderate sedation. During the perioperative period, tympanic temperatures, thermal comfort score, and shivering grade were recorded.

**Results:** Core temperature at the end of surgery did not differ significantly between the groups (36.4 ± 0.4 and 36.1 ± 0.7°C in the dexmedetomidine and propofol groups, respectively; P = 0.118). The lowest perioperative temperature, incidence and severity of perioperative hypothermia, thermal comfort score, and shivering grade did not differ significantly between the groups (all P > 0.05).

**Conclusions:** In patients undergoing spinal anesthesia with moderate sedation, the effect of dexmedetomidine on patients' core temperature was similar to that of propofol.

## Introduction

Inadvertent perioperative hypothermia (core temperature < 36.0°C) occurs frequently during regional and general anesthesia 1-4 and may lead to cardiac complications (e.g., arrhythmia or myocardial infarction), bleeding, need of blood transfusion, delayed postanesthetic recovery, impaired wound healing, wound infection, pressure ulcers, poor surgical outcomes, prolonged hospital stay, and increased medical costs 1,5,6. Thus, it is essential to prevent perioperative hypothermia.

Lower-extremity surgery can be performed under spinal anesthesia, and sedatives such as propofol and dexmedetomidine may also be used to relieve patients' anxiety and discomfort 7. Propofol can easily be titrated and has rapid onset and offset of action, while dexmedetomidine causes minimal respiratory depression and has anxiolytic and analgesic effects 8,9. However, propofol and dexmedetomidine cause varying degrees of impaired thermoregulation. Additionally, combined use of spinal anesthesia and sedatives increases the risk of hypothermia compared to spinal anesthesia alone 6.

Dexmedetomidine is a potent and highly selective α_2_-adrenoceptor agonist; at high plasma concentrations or after rapid intravenous administration, dexmedetomidine acts directly on postsynaptic vascular smooth muscle, resulting in vasoconstriction 10,11. In previous studies, dexmedetomidine increased systemic vascular resistance at plasma concentrations of 0.7-14.7 ng/mL 12, while propofol caused vasodilation in a dose-dependent manner 13. Therefore, we hypothesized that dexmedetomidine would be more advantageous than propofol in maintaining normal core temperature, because core-to-peripheral redistribution of body heat due to vasodilation at 30 min to 1 h after induction of general or neuraxial anesthesia is the primary cause of hypothermia 1. Previous studies have mainly evaluated differences in the efficacy of sedatives used during spinal anesthesia in terms of sedation, cardio-respiratory profile, and psychomotor performance 8,9. However, to date the effects of intraoperative administration of propofol and dexmedetomidine on thermoregulation in patients undergoing spinal anesthesia have not been compared. Therefore, we compared the core temperatures of patients who received dexmedetomidine or propofol for moderate sedation during orthopedic lower extremity surgery under spinal anesthesia.

## Materials and Methods

This prospective, randomized, controlled study was approved by Institutional Review Board (approval number: 2020-07-024) and registered at the Korea Clinical Research Information Service. Written informed consent was obtained from patients and/or their representative. The study protocol adhered to the Consolidated Standards of Reporting Trials (CONSORT) guidelines.

The study population included patients aged 19-70 years with an American Society of Anesthesiologists (ASA) Physical Status class of I-III who underwent elective orthopedic lower-limb surgery with an expected surgical duration of 30-120 minutes. We excluded patients who received general anesthesia or who had a preoperative core temperature < 36.0°C or > 37.5°C, morbid obesity (body mass index, BMI > 35 kg/m^2^), uncontrolled diabetes (A1C > 7.0%), hypothyroidism, abnormal coagulation, infection, or psychiatric or cognitive disease.

Patients were randomly allocated to the dexmedetomidine or propofol group at a 1:1 ratio using online randomization software (Researcher Randomizer; www.randomizer.org). In the preoperative holding area, the researcher who prepared the dexmedetomidine or propofol for administration opened an opaque, sealed envelope containing the patient's group assignment.

In accordance with our institutional protocol for general anesthesia, patients fasted for at least 8 hours preoperatively. The baseline core temperature was measured in the preoperative holding area using an infrared tympanic thermometer (Thermoscan IRT 4020; Braun GmbH, Kronberg, Germany), which was accurate to ± 0.2°C at temperatures of 35.5-42°C and ± 0.3°C at temperatures < 35.5°C.

In the operating room, before and during the spinal anesthesia, electrocardiogram, noninvasive blood pressure, and pulse oximetry were monitored. Spinal anesthesia was administered in the lateral decubitus position with the surgical site down. After the skin had been prepared and lidocaine injected, the dura was punctured at the L3-4 or L4-5 level using a 25-gauge Quincke needle. Once the clear cerebrospinal fluid was observed, 0.5% hyperbaric bupivacaine (10-12 mg) and fentanyl (10 μg) were injected. Anesthesia was considered adequate if the sensory block level was higher than T12 on pinprick test at 5 min after spinal anesthesia.

Following confirmation of adequate anesthesia, a forced-air warmer was set at 38°C (“medium” settings) and applied to the upper body. The core temperature was measured every 15 min until the end of the operation. In the dexmedetomidine group, an initial dose of 1 μg/kg of dexmedetomidine was administered over 10 min followed by a maintenance dose of 0.2-0.7 μg/kg/h. In the propofol group, a loading dose of 75 μg/kg of propofol was administered over 10 min followed by a maintenance dose of 12.5-75 μg/kg/min. In both groups, the sedative dose was titrated to achieve moderate sedation (Observer's Assessment of Alertness/Sedation, OAA/S scale score 3-4) 9. The OAA/S score and core temperature were evaluated every 15 min, and the infusion of sedatives was stopped when skin closure was started.

If the intraoperative systolic blood pressure decreased to < 90 mmHg or < 80% of the baseline value, 50-100 μg of phenylephrine, or in the event of bradycardia (heart rate < 50/min), 5-10 mg of ephedrine was administered. In the event of intraoperative bradycardia without hypotension, 0.5 mg of intravenous atropine was administered.

In the post-anesthesia care unit (PACU), the thermal comfort score (0 = completely uncomfortable; 10 = completely comfortable) was evaluated and the core temperature was measured every 15 min. Patients who complained of feeling cold or had a core temperature < 36°C received forced-air warming. The forced-air warmer was set at 43°C (“high” setting) but lowered to 38°C (“medium” setting) in the event of patient discomfort. Pain was treated with 0.5 μg/kg of fentanyl, shivering grade ≥ 2 (0 = no shivering; 1 = shivering localized to neck and thorax; 2 = shivering involving the upper extremities with or without shivering in the thorax; and 3 = shivering involving the entire body) was treated with 25 mg of meperidine, and nausea or vomiting was treated with 10 mg of metoclopramide.

Hypothermia was defined as tympanic temperature < 36°C and classified as mild (35-35.9°C), moderate (34-34.9°C), or severe (≤ 34°C) 2. The primary outcome was core temperature at the end of surgery. Secondary outcomes were perioperative lowest body temperature, incidence and severity of perioperative hypothermia (i.e., from preoperative holding area to PACU), thermal comfort score, shivering grade, and changes in core temperature during the perioperative period.

### Statistical analysis

The minimum difference required to detect perioperative temperature-related adverse outcomes is 0.5°C 3,6. To detect a 0.5°C (± 0.5°C) difference in core temperature at the end of surgery between two groups, with a power of 0.8 and an α-value of 0.05 (two-sided), 16 patients per group was required. Considering a dropout rate of 20%, 20 patients per group were recruited.

Data were analyzed using PASW Statistics software (version 18; IBM Corp., Armonk, NY, USA). Continuous data were analyzed using the Student's *t*-test or Mann-Whitney U test, as appropriate, and the distribution of data was assessed using the Kolmogorov-Smirnov test. Categorical variables were compared using the χ^2^ test, χ^2^ test for trends (linear-by-linear association), or Fisher's exact test, as appropriate. Changes in core temperature and OAA/S were analyzed using repeated-measures analysis of variance with Bonferroni correction. A two-sided P < 0.05 was considered significant.

## Results

**Fig 1:**
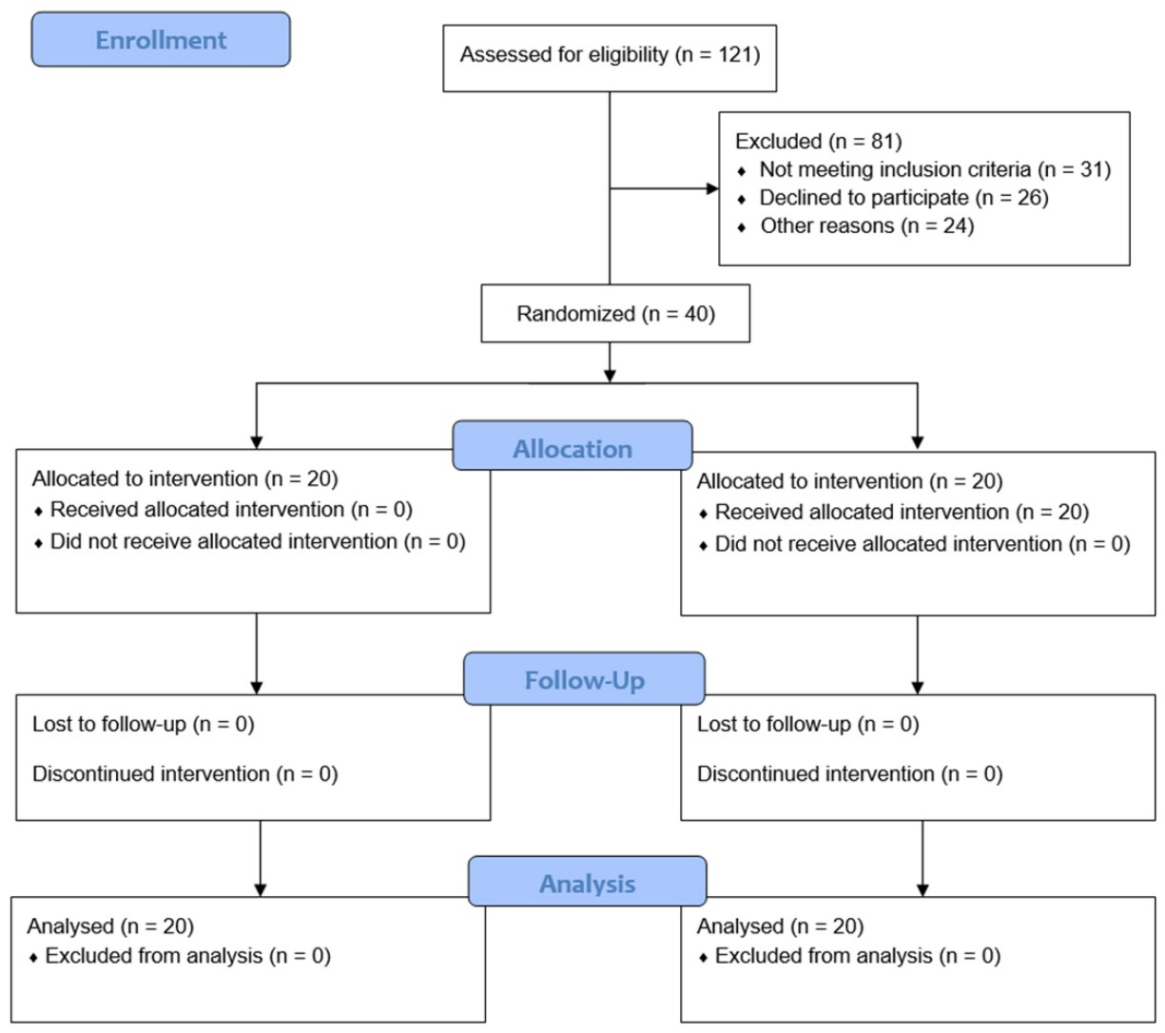
Flow chart. Group P, propofol group; Group D, dexmedetomidine group.

In total, 121 patients were assessed for eligibility; 81 patients were excluded because of age (< 19 or > 70 years; n = 31), lack of consent (n = 26), neuropsychological disease (n = 8), coagulopathy or bleeding diathesis (n = 7), infection (n = 4), uncontrolled diabetes (n = 3), or BMI > 35 kg/m^2^ (n = 2). Therefore, 40 patients were randomly allocated to the propofol or dexmedetomidine group (Fig. 1). The patient characteristics and baseline perioperative data of the two groups were similar (Table 1).

Table 2 lists perioperative body temperatures and outcomes. The preoperative baseline temperatures of the two groups were comparable. The primary outcome variable, i.e., core temperature at the end of surgery, did not differ significantly between the two groups (36.1 ± 0.7°C and 36.4 ± 0.4°C in the propofol and dexmedetomidine groups, respectively; mean difference -0.29; 95% confidence interval [CI] -0.65 to 0.08; P = 0.118). The lowest perioperative body temperature (P = 0.424), incidence and severity of perioperative hypothermia (P = 0.490 and P = 0.298, respectively), thermal comfort score at arrival in the PACU (P = 0.640), and grade of shivering (P > 0.999) also did not differ significantly between the groups.

**Fig 2:**
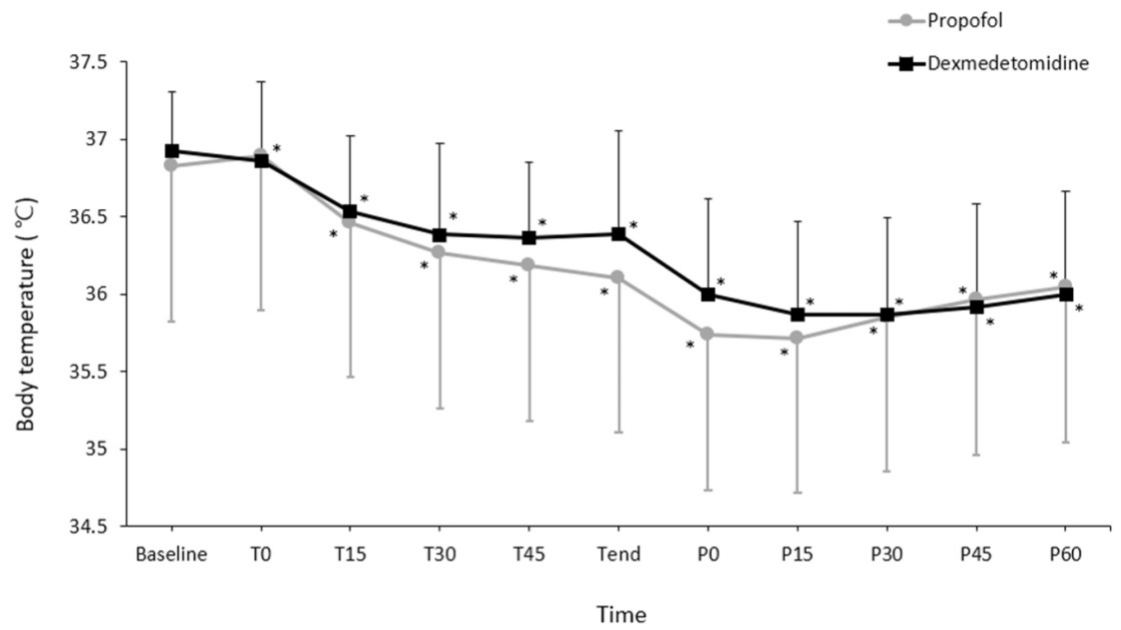
Changes in core temperature during the perioperative period. Values are presented as mean ± standard deviation. Baseline, arrival in the preoperative holding area; T0-45, immediately to 45 min after induction of spinal anesthesia (checked every 15 minutes); T_end_, at the end of surgery; P0-60, immediately to 60 min after arrival to the post-anesthetic care unit (checked every 15 minutes). ^*^P < 0.05, vs. baseline in each group (Bonferroni corrected). Core temperatures did not differ between groups at any time point (all P > 0.05, Bonferroni corrected)

**Fig 3:**
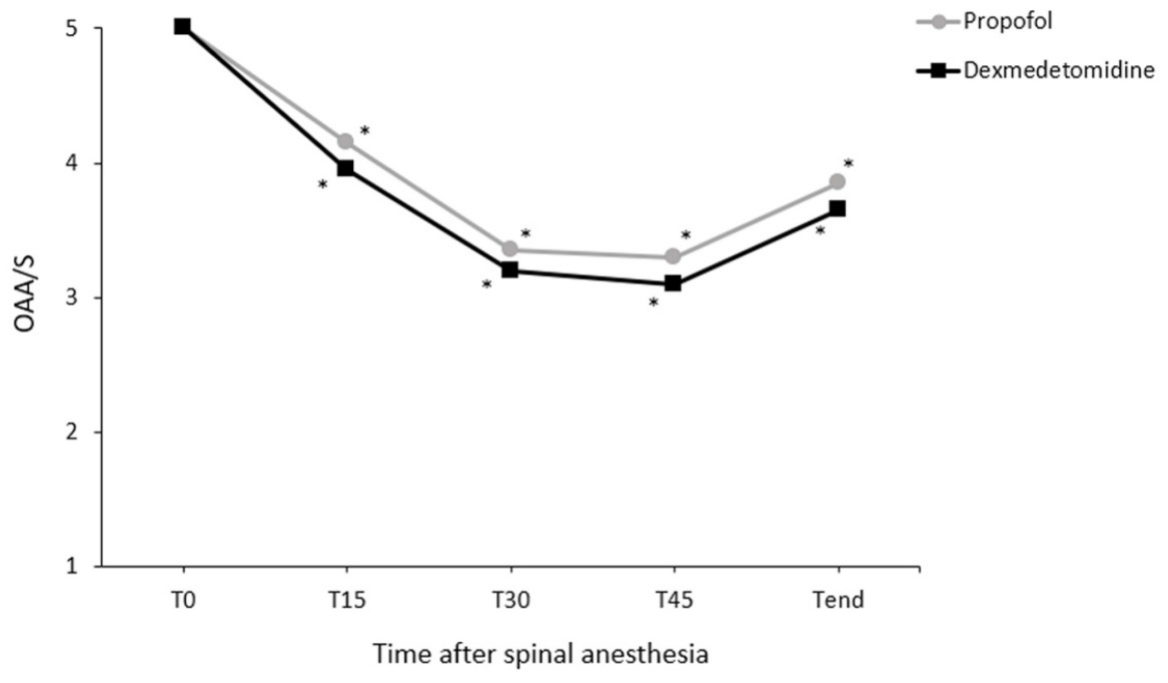
Changes in the Observer's Assessment of Alertness/Sedation (OAA/S) scale during the anesthesia. Values are presented as means. T0-45, immediately to 45 min after induction of spinal anesthesia (checked every 15 minutes); T_end_, at the end of surgery. ^*^P < 0.05, vs. baseline in each group (Bonferroni corrected).

Changes in core temperature during the perioperative period differed significantly between the groups (P = 0.048; Fig. 2). However, pairwise comparisons did not reveal significant intergroup differences at any time point. Changes in the OAA/S scores during spinal anesthesia did not differ significantly between the groups (P = 0.354; Fig. 3).

## Discussion

The objective of this study was to investigate the effects of dexmedetomidine and propofol on hypothermia during spinal anesthesia with moderate sedation. Core temperature at the end of surgery (the primary outcome), as well as the lowest perioperative body temperature, incidence and severity of perioperative hypothermia, thermal comfort score, and shivering grade (secondary outcomes), did not differ significantly between the two groups. Also, although changes in core temperature during the perioperative period showed a difference between the two groups, pairwise comparisons showed no intergroup difference at any measurement time point. These results imply that the effect of dexmedetomidine is similar to propofol in the prevention of hypothermia in patients receiving spinal anesthesia under moderate sedation.

The vasoconstrictive sympathetic response is responsible for heat preservation 6. In a previous study, administration of the pure α_1_-adrenergic agonist phenylephrine during surgery reduced the magnitude of redistribution hypothermia by maintaining precapillary vasoconstriction of blood vessels 14. Dexmedetomidine is an α_2_ agonist that has a sympatholytic effect by activating central α_2_ adrenoceptors and causes peripheral vasoconstriction through the peripheral vascular smooth muscle α_2_ adrenoceptors 9,10. We hypothesized that peripheral vasoconstriction due to dexmedetomidine would maintain the body temperature, but the results revealed similar clinical outcomes of dexmedetomidine and propofol administration.

Similar to general anesthesia, spinal anesthesia inhibits central thermoregulation. Additionally, spinal anesthesia prevents vasoconstriction in anesthetized regions by inhibiting the peripheral sympathetic motor nerves 6. Spinal-anesthesia-induced blockade of the preganglionic sympathetic fibers in anesthetized regions leads to vasodilation 15. Consequently, the vasoconstriction threshold of patients who receive spinal anesthesia decreases by approximately 0.6°C, and unwarmed anesthetized patients typically experience a decrease in core temperature of 1-2°C 5,6. Consistent with these reports, the lowest perioperative body temperature decreased by a mean of more than 1°C in both groups compared to the preoperative temperature. Additionally, the incidences of perioperative hypothermia were 75% and 65% in the propofol and dexmedetomidine groups, respectively, despite the intraoperative use of forced-air warming.

The use of sedatives in combination with spinal anesthesia may have affected the incidences of hypothermia mentioned above. Sedatives are used during spinal anesthesia for patient comfort, anxiolysis, cooperation, and reduction of the need of opioid analgesics 7,16. The use of sedatives during regional anesthesia significantly increases patient satisfaction and improves the quality of anesthesia and surgery 7. Despite these advantages, the use of sedatives during spinal anesthesia may further impair the perception of core cooling and autonomic protective responses against cold 6. To varying degrees, both propofol and dexmedetomidine inhibit the thermoregulatory center and sympathetic outflow, thereby lowering the threshold for vasoconstriction and shivering in a dose-dependent manner 5,6.

Intravenous administration of dexmedetomidine induces vasoconstriction within 3-5 min, followed by steady-state vasoconstriction for 10-15 min 10. However, the activation of endothelial nitric oxide synthase attenuates the vasoconstrictive effect of dexmedetomidine followed by vasodilation 17. Vasoconstriction is induced early after administration of high doses of dexmedetomidine, which is followed by delayed vasodilation once the drug plasma concentration decreases 10. Additionally, the cardiovascular effect of dexmedetomidine varies by concentration. At lower plasma concentrations, the plasma concentration of norepinephrine is reduced by more than 50%, resulting in a decrease in blood pressure, but peripheral vasoconstriction is not induced. However, dexmedetomidine increases blood pressure, systemic vascular resistance, and peripheral vascular resistance at plasma concentrations exceeding 1.9 ng/mL 12. Therefore, although the plasma concentration of dexmedetomidine was not measured, the concentration of dexmedetomidine used in the present study was probably insufficient to maintain adequate vasoconstriction to prevent hypothermia.

Another important factor affecting the incidence of hypothermia is intrathecal use of fentanyl and bupivacaine. Fentanyl is a highly ionized, lipophilic µ-receptor agonist that inhibits shivering by acting on the central thermoregulator and afferent thermal inputs in the spinal cord 18. Shivering is an unpleasant symptom that increases oxygen consumption, lactic acidosis, carbon dioxide production, and postoperative pain 1. Prevention of shivering with fentanyl improves symptoms but increases the risk of hypothermia due to inhibition of the thermoregulatory defense against cold 5,6,18. Therefore, the use of fentanyl may mask the direct and singular effects of drugs on hypothermia.

This study had several limitations. First, we used an infrared tympanic thermometer to estimate the core temperature. An infrared tympanic thermometer is easy to use in awake patients and is therefore commonly used in patients receiving regional anesthesia 4. However, the reliability of infrared tympanic thermometers is not clear 19. Standard core temperature can also be measured at the pulmonary artery, esophagus, or nasopharynx using a thermistor or thermocouple probe 6. However, these modalities are difficult to use in patients receiving regional anesthesia 20. Importantly, the measurement site has more influence on the precision and accuracy of temperature measurement than the measurement device 5. Additionally, measurements collected using an infrared tympanic thermometer have similar accuracy to those collected using pulmonary artery catheterization 21. Second, the doses of dexmedetomidine and propofol used in the present study were adjusted to provide moderate sedation. The sedative effect of dexmedetomidine is concentration-dependent: a plasma concentration of 0.2-2.0 ng/mL causes minimal or moderate sedation, whereas a plasma concentration > 2.0 ng/mL causes deep sedation 11,22. The hypotensive effect of dexmedetomidine is overwhelmed by the hypertensive effect of dexmedetomidine due to peripheral vasoconstriction at concentrations of 1.9-3.2 ng/mL 22. If the doses of the drugs were adjusted to achieve deep sedation, the doses of dexmedetomidine and propofol would be higher than those used in the present study, leading to different results. These limitations should be addressed in future studies. Third, dexmedetomidine generally showed a more favorable trend than propofol in maintaining normal body temperature, but there was no statistical difference between the groups. Future studies with large sample sizes may show the benefits of dexmedetomidine over propofol.

In conclusion, dexmedetomidine titrated to provide moderate sedation did not differ in terms of thermoregulatory end points, including core temperature at the end of surgery, incidence and severity of perioperative hypothermia, thermal comfort, and shivering severity, compared to propofol used to achieve a similar level of sedation in patients undergoing orthopedic lower limb surgery under spinal anesthesia. Therefore, in terms of prevention of perioperative hypothermia in patients undergoing surgery under spinal anesthesia with moderate sedation, dexmedetomidine and propofol use are associated with similar outcomes.

## Author Contributions

All of the listed authors were involved in the drafting of the work, approved the final manuscript, and agreed to be accountable for all aspects of this work.

1. Minhye Chang, Sung-Ae Cho

This author helped writing the manuscript, analyzing and interpretation of data.

2. Seok-Jin Lee, Choon-Kyu Cho, Young Seok Jee

These authors helped the acquisition, analysis, and interpretation of data.

3. Tae-Yun Sung

This author helped the conception and design of the study, statistical analysis and writing the manuscript.

## Figures and Tables

**Table 1:** Patient characteristics and perioperative data

	Group P (*n* = 20)	Group D (*n* = 20)	P
Age, y	59.7 ± 7.7	58.5 ± 12.5	0.718
Sex, male/female	11/9	9/11	0.527
Weight, kg	68.5 ± 11.2	66.0 ± 12.4	0.518
Height, cm	160.7 ± 9.4	161.4 ± 10.1	0.833
Body mass index (kg/m^2^)	26.6 ± 4.5	25.2 ± 3.5	0.293
ASA physical status (I-III)	2/17/1	2/18/0	0.651
Ambient operating room temperature, °C	21.8 ± 0.9	21.7 ± 1.3	0.855
Sensory block level	T6 (T5-T9)	T6 (T4-T10)	0.968
Ephedrine, mg	10.3 ± 10.6	11.7 ± 12.4	0.683
Phenylephrine, μg	0 (0-800)	0 (0-1500)	0.883
Atropine, mg	0 (0-1)	0 (0-1)	0.063
Fluids, mL	678.5 ± 497.7	477.5 ± 414.4	0.173
Estimated blood loss, mL	30 (5-100)	30 (5-150)	0.355
Duration of surgery	84.8 ± 42.1	83.5 ± 19.4	0.905
Duration of anesthesia	119.8 ± 41.6	118.5 ± 20.3	0.905

Values are presented as mean ± standard deviation, median (range) or number. Group P, propofol group; group D, dexmedetomidine group; ASA, American Society of Anesthesiologists.

**Table 2:** Perioperative patient temperatures and outcomes

	Group P (*n* = 20)	Group D (*n* = 20)	Mean difference (95% CI)	P
Preoperative temperature, °C	36.8 ± 0.4	36.9 ± 0.3	-0.08 (-0.3, 0.14)	0.460
Temperature at the end of surgery, °C	36.1 ± 0.7	36.4 ± 0.4	-0.29 (-0.64, 0.08)	0.118
The lowest temperature, °C	35.6 ± 0.6	35.7 ± 0.4	-0.13 (-0.44, 0.19)	0.424
Incidence of hypothermia	15 (75%)	13 (65%)	10% (-17.6%, 35.7%)	0.490
Severity of hypothermia				0.298
Normothermia (≥ 36.0°C)	5 (25.0%)	7 (35.0%)	NA	NA
Mild (35-35.9°C)	12 (60.0%)	12 (60.0%)	NA	NA
Moderate (34-34.9°C)	3 (15.0%)	1(5.0%)	NA	NA
Severe (≤ 34°C)	0	0	NA	NA
Thermal comfort score, 0-10	9 (6-10)	10 (4-10)	NA	0.640
Shivering grade (0-3)	17/3/0/0	18/2/0/0	NA	> 0.999

Values are presented as mean ± standard deviation, median (range), or number. Group P, propofol group; Group D, dexmedetomidine group; CI, confidence interval; NA, not applicable.
